# Modelling age-friendly environment for social connectedness: a cross-sectional study

**DOI:** 10.12688/f1000research.73032.1

**Published:** 2021-09-22

**Authors:** Lein Shi Ying, Lai Ming Ming, Lau Siok Hwa

**Affiliations:** 1 Faculty of Management, Multimedia University, Persiaran Multimedia, Cyberjaya, Selangor, 63100, Malaysia; 2 Faculty of Business, Multimedia University, Jalan Ayer Keroh Lama, Bukit Beruang, Melaka, 75450, Malaysia

**Keywords:** age-friendly, social connectedness, aging, Malaysia, environment

## Abstract

**Background: **The increase in aged populations in Malaysia has brought unprecedented challenges to families, policy makers, scholars, and business organisations.  This paper adapted the WHO 2007 framework of features of age-friendly cities to examine age-friendly environment constructs and their linkages with social connectedness from the perspective of Malaysian middle-aged and older adults caring for themselves.

**Methods: **A face-to-face cross-sectional survey was conducted on 402 middle-aged and older adults caring for themselves across selected states in west Malaysia, selected via purposive sampling. Firstly, features of age-friendly cities were explored through exploratory factor analysis involving 82 respondents. Subsequently, structural equation modelling was performed, involving 320 respondents.

**Results: **The results indicated that the constructs of an age-friendly environment were built environment, community support and health services, civic participation, and employment as well as communication and information. The structural model provided evidence that implementing age-friendly initiatives relating to built environment, community support and health services, civic participation and employment as well as communication and information enables the ageing population to improve their connectedness with society. These findings supported the ecological theories, agreeing that age-friendly environments help middle-aged and older adults caring for themselves to increase their adaptability and reduce perceived pressure from the environment. This result was in line with the current literature in which an age-friendly environment is a form of support and an enabling environment to cultivate positive social relationships and connectivity.

**Conclusions: **Creating an age-friendly environment that supports active and healthy living for middle-aged and older adults caring for themselves allows them to continue to share their experiences, knowledge, and wisdom. This is helpful and beneficial for societal well-being and economic development as well as for the future generations.

## Introduction

Malaysia is expected to become an aged nation by 2030 where 15.3%, approximately 5.8 million of its population, would comprise of individuals aged 60 and above according to the Department of Statistics, Malaysia. ^1^ Malaysia is categorised as one of the nations that is ageing at the fastest pace because Malaysia would transform into an aged nation within 20 years, as individuals aged 60 and above only comprised of 7.6% of its population in year 2010. ^2^ The main problems faced by ageing adults in Malaysia are illness, deteriorating physical functioning, existing building and facilities not being age-friendly designed, as well as less social interactions and feeling less connected to others. ^3^

According to The World Factbook, the majority of Malaysians (77.7%) live in urban areas. Urbanisation and population ageing have become issues for healthcare-related personnel, researchers, and town planners in developing healthy and liveable environments for the elderly. ^4^ The threats found in urban areas are harmful to social well-being such as environmental pollution, tobacco and alcohol abuse, malnutrition, lack of physical activities, inaccessible transportation, poor infrastructure, and inadequate healthcare services. ^5^ Furthermore, new buildings and most of the old buildings in Malaysia were built without meeting the statutory guidelines for disabled persons. ^6^ The development of an age-friendly environment is essential because the current environment is built without taking into considerations the needs of people of all ages. ^7^

Having the opportunity to deepen existing relationships and social connectedness with others is important for those who are ageing. ^8^ Relationships become gradually more important when an individual ages; through an age-friendly environment initiative, one can improve one’s social connections and receive adequate social support, which is beneficial for maintaining health and well-being. ^9^ In order to improve the well-being of the ageing population, an age-friendly environment should be developed to support social connectedness, participation, and integration among the elderly. ^10^

In acknowledging the growth of the ageing population worldwide, the World Health Organisation (WHO) introduced two policy frameworks, namely, active ageing ^11^ and global age-friendly cities. ^12^ WHO (2007, p. 1) ^12^ defined an age-friendly city as one that “encourages active ageing by optimizing opportunities for health, participation, and security in order to enhance quality of life” of elderly individuals. An age-friendly community helps the elderly “to maintain social connectedness while deepening existing relationships” (Emlet & Moceri, 2012, p. 2). ^10^

Empirical studies of the age-friendly concept are few. ^13^ Despite Malaysia being predicted to become an aged nation by 2030, studies conducted in relation to the ageing of the population are relatively limited compared to developed countries. There is an urgency to restructure policies for the ageing population that includes local factors that address the forthcoming health and social needs. ^14^ The ageing of the population in Malaysia has created unprecedented challenges to policy makers, scholars, and business organisations as Malaysia will become an aged nation by 2030.

In an initiative to make environments better for the elderly and to accommodate their needs, the WHO Global Network of Age-friendly Cities and Communities was formed to facilitate mutual communication, discussions, and learning among countries, cities, and communities globally. Many developed countries, cities, and communities joined the network and adopted the global age-friendly cities guide. ^12^ To date, the network consists of 847 cities and communities across 41 countries, which include more than 230 million people, and the network is expanding. Taiping city in Perak, Malaysia was chosen for the two-year age-friendly city pilot project in August 2019 and is on track to achieve age-friendly city status.

It should be pointed out that Malaysia has not adopted the WHO (2007) global age-friendly cities guide. ^12^ Hence it is important to identify the constructs of age-friendly environments and subsequently examine the relationships between age-friendly environments and social connectedness from the perspectives of middle aged and older adults caring for themselves in Malaysia. This study empirically modelled an age-friendly environment and social connectedness model through structural equation modelling (SEM). SEM provides higher accuracy in predicting the research model as validity of the model is statistically evidenced through the assessment of goodness-of-fit indices. ^15^

This study aims to empirically identify the significant constructs of age-friendly environments by adapting the WHO (2007) global age-friendly cities ^12^ guide as well as examining the relationship between age-friendly environments and social connectedness by bridging the literature gap as suggested by Menec *et al.* (2011) ^16^ and Emlet and Moceri (2012). ^10^ This is among the first few attempts to model an age-friendly environment by identifying its significant constructs and subsequently linking to social connectedness from the perspective of middle-age and older adults caring for themselves in a developing country. The proposed research model is important and adds empirical evidence on the readiness and preparation of Malaysia for becoming an aged nation by 2030.

### Literature review

An age-friendly environment offers a comprehensive “age-integrated as well as age-targeted” initiative that helps the ageing adults to improve their social connectedness and self-worth ^12^ (WHO, 2007, p. 72). The concept of social connectivity is an indispensable advantage of age-friendly environments given that an age-friendly environment forms a mutual connection between the ageing adults and their environment. ^16^ The literature suggests that local authorities and practitioners should focus on neighbourhood design and establish community-based interventions to improve social connectedness and social participation among the elderly. ^17^^,^^18^

Lawton’s ecological theory of ageing (Nahemow & Lawton, 1973) ^19^ and Bronfenbrenner’s ecological theory (1977) ^20^ are widely used to address the adaptation between environment and humans, whereby there should be a balance between both. ^19^^,^^20^

The environment can bring certain pressures or hassle to daily living as an individual ages, which would create inconveniences for them. ^21^ According to the ecological theory of Lawton (1974), ^21^ there are two main causes for the decrease in capacity as one ages, namely perceived pressure from the environment and weakening of bio-physical functioning.

Bronfenbrenner (1977) ^20^ explained that the ecological environment can be visualised as a set of nested environmental structures, which are arranged at each inclusion of another and interact bi-directionally. The structures are the microsystem, mesosystem, exosystem and macrosystem. Microsystem refers to interactions between an individual and their immediate environment such as family, home, friends, and workplace. Mesosystem refers to the interconnectivity of an individual with the interaction of multiple immediate environments. According to the Bronfenbrenner’s ecological theory (1977), ^20^ individuals interact with the different environment structures in daily living. The ecological frameworks focus on the interconnectivity between humans and varied social and physical environments. ^22^ Both the ecological frameworks emphasise the vitality of the social aspect of an environment. ^20^^,^^23^

Lui *et al.* (2009) ^24^ and Menec *et al.* (2011) ^16^ indicated that elderly people would like to remain active and be involved in community activities in their silver days. An age-friendly environment supports and enhances social connectivity among the ageing population.

In the application of ecological frameworks to conceptualise an age-friendly environment, the environment should be dynamic to accommodate the changes in physical and cognitive functioning as well as social capacity as people grow older. ^10^ Furthermore, social inclusion is a significant component in addressing age-friendly environments and could be improved by removing the obstacles brought by elements of the physical and social environments. ^25^

Feldman and Oberlink (2003) ^26^ are amongst the pioneers of conceptualisation of age-friendly environments. They conducted focus group interviews involving adults aged 35 and above and community leaders from the United States. The study found four indicators of age-friendly environments, namely identifying basic necessities, promoting social and civic engagement, maximising independence for frail and disabled elderly and optimising physical and mental functioning and well-being. The study concluded that financial security, health and healthcare, social connections, housing and supportive services, transportation and safety were important factors for the elderly to age-in-place.

Subsequently, WHO (2007) ^12^ conducted focus group interviews to examine age-friendly city features from a wider perspective involving 1,485 elderly people aged 60 and above, 250 caregivers, and 515 service providers across 33 cities across continents worldwide. They found eight features that made a city age-friendly, which were outdoor spaces and buildings, transportation, housing, social participation, respect and social inclusion, civic participation and employment, community support and health services, as well as communication and information. On the other hand, Alley *et al.* (2007) ^27^ studied elder-friendly communities using qualitative methods. They found that the significant criteria of elder-friendly communities were accessible and inexpensive transportation, safe residences, healthcare, safety, and being involved in the community.

Richard *et al.* (2009) ^17^ examined the associations between neighbourhood features and social participation among 282 urban elderly people in Canada through a survey. The results found that social participation had positive implications for their health. Hence, they suggested that the urban and local community authorities build a supportive neighbourhood that promoted social interaction and participation among the elderly.

Menec *et al.* (2011) ^16^ conceptualised the age-friendly community based on the WHO’s 2007 age-friendly city model ^12^ and ecological theory. Their study proposed seven important domains, namely physical environment, housing, social environment, opportunities for participation, informal and formal community support and health services, transportation and communication and information. They also suggested that the concept of social connectivity served as a basic support to age-friendly communities. Subsequently, Menec *et al.* (2013) ^13^ explored the characteristics of age-friendliness in Canada through a survey of 1,373 individuals. A larger percentage of the respondents scored highly on social age-friendly domains, such as social environment, participation opportunities, health-care services, and communication and information, in comparison with physical domains. Hence, they found that age-friendly domains in the social environment were easier to implement than in the physical environment.

From the Asian perspective, Kadoya (2013) ^28^ explored the barriers faced by the elderly when interacting with society through their participation in local community programmes in Japan and found that the ability to be mobile affected their social interaction. Hence, he concluded that social inclusion was an important factor for age-friendly cities. Lai *et al.* (2016) ^29^ studied the WHO (2007) age-friendly ^12^ environment features and linked them with active ageing. The study surveyed 211 informal caregivers and 402 adults who cared for themselves aged 45 and above in Malaysia through purposive sampling. They found that outdoor spaces and buildings, transportation, and housing as well as community support and health services were significant age-friendly environment features. The findings implied that the development of physical environment features such as a comprehensive public transportation system, elderly-friendly housing, outdoor spaces and buildings as well as medical and healthcare services should be carried out before social environment development.

Franke *et al.* (2013) ^18^ conducted a qualitative study to investigate the main areas that promoted physical activity among the elderly who were ageing actively in Canada. These areas were resourcefulness, social connections, and built environment. The study suggested that new policies addressing social connectedness and accessibility to social and physical environments were required to enhance and preserve independence and mobility among the elderly.

Scharlach and Lehning (2013) ^25^ reviewed and described the various age-friendly efforts implemented in the United States, which helped to enhance social inclusion among the elderly from the perspective of physical and social environments. An environment that enabled the elderly to be mobile and with an accessible transportation system would help to promote social inclusion. In general, social inclusion could be improved by removing the hurdles found in the physical and social environments.

Ruza *et al.* (2015) ^30^ compared the perspectives of age-friendliness among professionals and local community residents via focus group interviews. Both the professionals and local groups agreed that community and health services had the greatest significance in establishing age-friendliness of a city. Meanwhile, communication and information as well as civic participation and employment were found to have the least significance.

Rogers and Mitzner (2017) ^31^ conducted a study to predict the personality, needs, and wants of the future elderly of 2050 by taking into account their independence, health, well-being, and social connectedness. There was a similarity between the future and current elderly; they wished to prolong their independence. Social connectedness and social activities were essential for the general well-being of older adults. Therefore, being socially connected with family, friends, and neighbours helped ageing adults to improve their life satisfaction.

**Figure 1.**
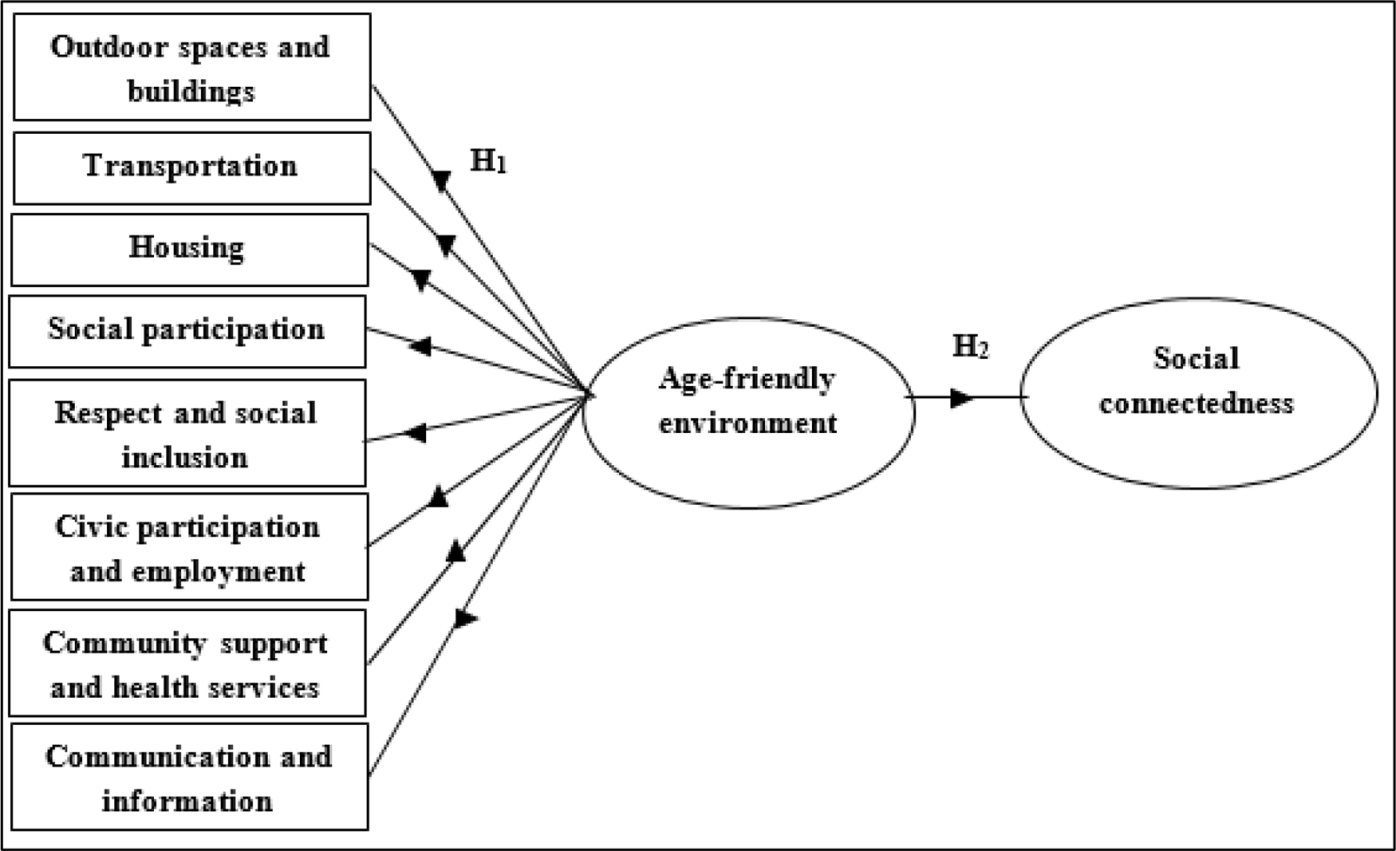
Research model of age-friendly environment and social connectedness.

To bridge the research gap and supported by ecological theories, this study empirically examines the constructs of age-friendly environments and proposed positive relationships between an age-friendly environment and social connectedness from a developing country perspective. Figure 1 illustrates the proposed research model of age-friendly environments and social connectedness; and the two hypotheses are as follows:

H _1_: Outdoor spaces and buildings, transportation, housing, social participation, respect and social inclusion, civic participation and employment, community support and health services, as well as communication and information will be confirmed as constructs of an age-friendly environment.

H _2_: An age-friendly environment is positively related to social connectedness.

This study examines eight features of age-friendly environments and social connectedness. These constructs are reflective models. As a result, the eight features form a latent variable of age-friendly environments. The latent construct of age-friendly environments is identified by a set of indicators. In short, the eight constructs of age-friendly environments are the indicators of age-friendly environments. Hence, an age-friendly environment is a formative model in which the valid constructs are confirmed through second-order confirmatory factor analysis (CFA).

## Methods

### Ethics statement

This research received approval (number EA1842021) from Research Ethics Committee of Multimedia University. Both informed and written consent were obtained from all participants prior to initiating the survey. When the respondents verbally agreed to participate, the questionnaire was given to them. The respondents who agreed to participate were required to tick on the column “If agree, please proceed to the next page” in the questionnaire.

### Study design and population

This study employed a cross-sectional survey method to examine the research model of age-friendly environments and social connectedness. In line with the prior studies by Feldman and Oberlink (2003), ^26^ Emlet and Moceri (2012), ^10^ and Menec *et al.* (2013), ^13^ middle-aged adults were used as targeted respondents. Middle-aged and older adults have witnessed death in their family and of their peers; thus, they may be compelled to improve their quality of life while they still have time. ^32^ Perceptions of age-friendly environments from middle-aged adults are helpful as they portray the future needs of the elderly, which is beneficial for policy planning and enables them to voice their opinion for the future community in which they would age. ^10^

Self-care refers to the ability of “people looking after themselves” (WHO, 2002, p. 37). ^11^ By adapting the definition by Levinson (1986), ^33^ middle-aged adults were individuals aged 45 to 65 years old, while older adults were individuals aged 65 and above. ^1^ This study identified targeted respondents as middle-aged adults and older adults undertaking self-care, who were individuals aged 45 and above and have the ability to take care of themselves.

Purposive sampling was used to collect data from respondents based on highly populated Malaysian states with high urbanisation levels and old-age dependency ratios. Hence the data was collected from Penang, Perak, Selangor, Wilayah Persekutuan Kuala Lumpur, Negeri Sembilan, Melaka, and Johor states in Malaysia. Since targeted respondents are middle-aged and old-age adults caring for themselves, places where many ageing adults are found or places which have high level of ageing adults’ activities were identified. The main research places of this study were the “Pusat Aktiviti Warga Emas (PAWE)”, or senior activity centers, in Pulau Pinang, Perak, Selangor, Wilayah Persekutuan Kuala Lumpur, Negeri Sembilan, Melaka, and Johor states. These senior activity centers provide a place where the elderly can gather and interact with one another. They are supported and governed by the Department of Social Welfare in Malaysia.

### Sample size

The study collected data from 402 self-care middle-aged and older adults, which fulfilled the minimum sample size as suggested by G*Power 3 statistical software. The criteria were set by employing *a priori* power analysis with medium effect size, alpha at 0.05, power at 0.8, as well as eight constructs. ^34^

The constructs of an age-friendly environment were adapted from the “checklist of essential features of age-friendly cities” (WHO, 2007b), which had not been empirically tested. Hence, the constructs of an age-friendly environment were firstly explored through exploratory factor analysis. G*power 3 statistical software suggested the sample size N = 52 is required for regression analysis through *a priori* power analysis by considering a large effect size, alpha of 0.05, power of 0.8, and eight constructs (Faul et al., 2009). ^34^

In performing the exploratory factor analysis, the study first selected 82 odd samples which comprised approximately 20% of the total sample size of 402. This sample, N = 82 fulfilled the minimum sample size required to analyse eight constructs based on *a priori* power analysis of large effect size, significance level at 0.05 and power of 0.8. Hence, the remaining sample of 320 was used to create the research model using structural equation modelling.

### Study instrument

The survey had three sections, namely demographic variables, age-friendly environment, and social connectedness. Demographic variables examined respondents’ age, gender, nationality, race, and educational background. The age-friendly measurement items were developed by adapting the indicators of activity-friendly communities, ^35^ checklist of essential features of age-friendly cities, ^36^ and information, communication, and technology (ICT) scale by Cotten, Anderson, and McCullough (2013). ^37^ The measurement items were examined using a five-point Likert scale ranging from 1 “very unimportant” to 5 “very important” to evaluate the level of importance of age-friendly environment features as perceived by the respondents. In examining social connectedness among respondents, the study adapted the social connectedness scale by Lee and Robbin (1995). ^38^ This is a self-rated scale of emotional distance or connectedness between an individual and others in a society. In this study, the scale was evaluated through a five-point Likert scale that ranged from 1 “strongly disagree” to 5 “strongly agree”. Higher scores indicate greater social connectedness as well as freedom from social isolation. The original items were phrased negatively; therefore, the items were re-coded for analysis.

The questionnaire ^54^ was developed in English and translated into Malay and Chinese using the direct translation method because this is an easy and simple procedure. The translation process began with direct translation of the questionnaire from English to the Malay and Chinese by language experts. The Malay and Chinese versions were reviewed and verified by a group of reviewers and researchers involved in this study who were familiar with English. The direct translation method can be improved through review by independent judges. ^39^ It is worth noting that standard procedures for translating measurement items had yet to be established. ^39^ There is “no empirical evidence in favour of one specific method” in translating measurement items (Acquadro *et al.*, 2008, p. 509). ^40^

The pilot study was conducted in Melaka, employing purposive sampling. The sampling location areas included local community areas such as parks, religious centres, and informal gatherings of middle-aged and older adults in residential areas. The pilot study was conducted in December 2013 among 30 middle-aged and older adults aged 45 and above who cared for themselves. Cronbach’s alpha reliability test was conducted to examine the reliability of the measurement items. The results showed that all variables were valid, indicated by a Cronbach’s alpha coefficient of more than 0.7. No major changes were made to the survey following the pilot study.

### Data collection

The actual data collection began from January 2014 until September 2014 and 402 responses were collected from middle-aged and older adults caring for themselves in the seven Malaysian states. Approval was sought from the Department of Social Welfare to allow the collection of data at senior activity centers in the selected states of Malaysia. It should be pointed out that the study only managed to collect 272 responses through face-to-face surveys administered by a graduate research assistant in the senior activity centers. These 272 responses did not achieve the minimum sample size required. Therefore, data was supplemented with data gathered from local community areas; for example, informal gatherings of middle-aged and older adults at religious places such as mosques, temples, or churches as well as gatherings of informal caregiver support groups of the Alzheimers Disease Foundation Malaysia in Petaling Jaya and Johor Bahru cities in Malaysia.

### Analysis

The Cronbach’s alpha test of reliability was conducted to examine the reliability of measurement items. Next, the 37 measures of age-friendly environments were explored through exploratory factor analysis (EFA) performed on 82 responses. EFA was conducted through principal axis factoring and promax rotation. Two-step structural equation modelling (SEM) was employed to examine the research model, which began with the specification of CFA and proceeded with structural model development. This study employed SPSS version 22 and Analysis of Moment Structures (AMOS) version 21.

The maximum likelihood method of estimation was utilised as it is the most commonly used method of estimation. ^41^ This estimation method measures all the variables concurrently at a point in time and provides the most accurate estimation with the least variance when data is normally distributed. ^42^ A common alternative to examine the normality of data is through monitoring the value of skewness and kurtosis and can be determined by the sample size. For a sample size greater than 300, “an absolute skew value larger than 2 or an absolute kurtosis larger than 7” are indicative that the data is not normally distributed (Kim, 2013, p. 53). ^43^

CFA allows the assessment of model goodness of fit and model validity. Model goodness of fit is indicated by insignificant p-value, normed chi-square value (CMIN/df) lower than 3, expected cross validation index (ECVI) near zero, standardised root mean residual (SRMR) lower than 0.08, root mean square error of approximation (RMSEA) lower than 0.07 with insignificant p-value, followed by goodness-of-fit index (GFI), adjusted GFI (AGFI), normed fit index (NFI), comparative fit index (CFI), and Tucker Lewis index (TLI) above 0.9. ^44^^-^^46^

Construct validity of the specified model was examined through convergent validity and discriminant validity. The three criteria of convergent validity are standardised factor loadings, average variance extracted (AVE), and reliability. ^44^ A measurement model achieved convergent validity when standardised factor loadings exceed 0.5 and preferably 0.7, AVE more than 0.5, as well as composite reliability (CR) larger than 0.7. ^44^ Discriminant validity was determined using the method proposed by Fornell and Larcker (1981). ^47^ Discriminant validity is achieved when AVE is larger than the value of squared correlation between constructs. When the constructs satisfy the model goodness of fit and validity assessment, a structural model for hypothesis testing can be specified. In this study, a structural model was specified by linking age-friendly environments and social connectedness.

## Results

A total of 402 middle-aged and older Malaysian adults caring for themselves participated in the study. The sample had a greater proportion of female participants than male; 274 (68.2%) female and 128 (31.8%) male. ^55^ The sample was evenly divided between 212 (52.7%) middle-aged adults and 190 (47.3%) older adults. The sample consists of a large number of Chinese respondents (240, 59.7%) and married respondents (260, 64.7%). The majority of the respondents (278, 69.2%) were not highly educated and their highest level of education was either primary level, secondary school level, or had attained Sijil Pelajaran Malaysia (SPM). An individual who has completed their secondary school education is awarded with Sijil Pelajaran Malaysia (SPM). ^48^ Results of demographic profiles are presented in Table 1.

**Table 1.** Demographic profile of respondents.

Demographic characteristics	N = 402	Demographic characteristics	N = 402
**Location**		**Ethnicity**	
Pulau Pinang	57 (14.2)	Malay	141 (35.1)
Perak	50 (12.4)	Chinese	240 (59.7)
Selangor	45 (11.2)	Indian	20 (5.0)
Wilayah Persekutuan Kuala Lumpur	77 (19.2)	Portuguese	1 (0.2)
Negeri Sembilan	58 (14.4)	**Marital status**	
Melaka	57 (14.2)	Single	38 (9.5)
Johor	58 (14.4)	Married	260 (64.7)
**Gender**		Widowed	96 (23.9)
Male	128 (31.8)	Divorced	8 (2.0)
Female	274 (68.2)	**Highest education level attained**	
**Age**		No formal education	47 (11.7)
45.1 to 55	66 (16.4)	Primary school	131 (32.6)
55.1 to 65	146 (36.3)	Secondary school	82 (20.4)
65.1 to 75	123 (30.6)	SPM	65 (16.2)
75.1 to 85	61 (15.2)	STPM/Diploma	38 (9.5)
≥85.1	6 (1.5)	Degree	32 (8.0)
		Postgraduate	7 (1.7)
**Total**	**402 (100.0)**	**Total**	**402 (100.0)**

Note: Percentages are in parentheses. SPM, Sijil Pelajaran Malaysia; STPM, Sijil Tinggi Persekolahan Malaysia.

Cronbach’s alpha test was performed to examine the reliability of the variables. An item from the housing construct was removed to improve the coefficient. Variables were reliable given that the value of Cronbach’s alpha coefficient for each variable was larger than 0.7, as shown in Table 2.

**Table 2.** Reliability analysis of age-friendly environment and social connectedness measures.

Variables	Cronbach’s alpha coefficient
**Age-friendly environment features**	
a) Outdoor spaces and buildings	0.92
b) Transportation	0.87
c) Housing	0.91
d) Social participation	0.84
e) Respect and social inclusion	0.89
f) Civic participation and employment	0.87
g) Community support and health services	0.95
h) Communication and information	0.98
**Social connectedness**	0.95

Subsequently, EFA through the principal axis factoring method and promax method of rotation was performed on 82 responses. A clean factor structure was achieved with four constructs, namely communication and information, built environment, community support and health services, and civic participation and employment. The result of the Kaiser-Meyer-Olkin (KMO) test was 0.84 and Bartlett’s test of sphericity was significant with p = 0.00. Pattern matrix analysis showed that factor loadings all exceeded 0.5, indicating that the analysis of factor structure was valid. The final factor structure suggested that the four factors be retained as each had eigenvalues higher than one. The results of EFA are shown in Table 3.

**Table 3.** Results of exploratory factor analysis of the age-friendly environment features (N = 82).

Age-friendly environment features	Pattern matrix			
	1	2	3	4
1. Communication and information				
a) Increases the quantity of my communication with others	0.95			
b) Contributes to my ability to stay in touch with people I know	0.95			
c) Makes me feel less isolated	0.94			
d) Makes it easier to meet new people	0.92			
e) Makes it easier for me to reach people	0.91			
f) Helps me feel more connected to friends and family	0.84			
2. Built environment				
a) Drop off and pick up areas are available and conveniently located		0.87		
b) Presence of bicycle lanes and walking trails		0.76		
c) Public transportation is accessible by everyone including people with disabilities		0.72		
d) Outdoor safety is promoted by good street lighting during the night		0.71		
e) Taxis have discounts for elderly people		0.71		
f) Public transportation is available for elderly people to reach key destinations		0.70		
g) Public toilets are conveniently accessible		0.68		
h) Presence of traffic signals with adequate time for crossing for elderly people		0.58		
3. Community support and health services				
a) Affordable physiotherapy services are offered to restore elderly health			0.91	
b) Health services are conveniently reachable			0.88	
c) Affordable medical care services are available for elderly people			0.82	
d) Adequate range of services are offered in the community to promote elderly well-being			0.58	
4. Civic participation and employment				
a) Flexible paid opportunities for elderly people to work				0.86
b) Elderly people have flexible voluntary options				0.84
c) Workplaces are adapted to meet the needs of elderly people with disabilities				0.71
Kaiser-Meyer-Olkin	0.84			
Eigenvalue	9.70	3.32	2.02	1.20
Percentage of variance (%)	42.77	14.16	7.55	4.19
Percentage of cumulative variance (%)	42.77	56.93	64.48	68.67

The EFA results showed that three features of age-friendly environment, namely outdoor spaces and buildings, transportation, and housing were grouped and explained as one single factor, which the study renamed as built environment. It was possible to re-classify these three features as a single factor given that they are the “key features of physical environment” (WHO, 2007, p. 9). ^12^ This study named the combination of the three features as built environment because built environment comprehensively addresses the features of outdoor spaces and buildings, transportation as well as housing. ^27^

At the same time, two features, namely respect and social inclusion as well as social participation, were removed as these items had cross-loading with other variables. WHO (2007, p. 9) identified respect and social inclusion, social participation, and civic participation and employment as “social environment and of culture that affect participation and mental wellbeing”. ^12^ Social participation as well as civic participation and employment were re-grouped and explained as opportunities for participation because they were related to participation of the ageing adults in social activities. ^16^ Civic participation and employment was found to be more practical in the context of Malaysia in addressing social participation among the ageing adults.

Subsequently, first-order CFA of age-friendly environment and social connectedness features was carried out with 27 items. To examine the correlation among the constructs, they were allowed to covary. Overall first-order CFA of age-friendly environment and social connectedness features allowed the evaluation of convergent validity and discriminant validity.

The initial model did not achieve satisfactory model fit. Therefore, model respecification was performed by examining modification indices as standardised factor loadings had exceeded 0.5. Items with high modification indices were removed or allowed to co-vary in the model. This is because the items that highly explain a construct should be retained, which helps to improve the accuracy of a specified model. ^49^ A satisfactory first order CFA of age-friendly environment and social connectedness features was achieved with a model fit of CMIN/df = 1.19, GFI = 0.96, AGFI = 0.94, RMSEA = 0.02, SRMR = 0.03, NFI = 0.98, TLI = 0.99, CFI = 0.99 and ECVI = 0.62. The results of CFA of age-friendly environment and social connectedness features as well as convergent validity are presented in Table 4.

**Table 4.** Result of confirmatory factor analysis of age-friendly environment and social connectedness features.

Age-friendly environment constructs	Convergent validity			
	Std. factor loadings ^**a**^	AVE ^**b**^	CR ^**c**^	Cronbach’s alpha
1. Communication and information		0.89	0.96	0.96
a) Makes it easier for me to reach people	0.92			
b) Contributes to my ability to stay in touch with people I know	0.97			
c) Makes me feel less isolated	0.94			
2. Built environment		0.67	0.86	0.86
a) Drop off and pick up areas are available and conveniently located	0.76			
b) Public transportation is accessible by everyone including people with disabilities	0.84			
c) Public toilets are conveniently accessible	0.86			
3. Community support and health services		0.88	0.96	0.94
a) Affordable physiotherapy services are offered to restore elderly health	0.97			
b) Affordable medical care services are available for elderly people	0.91			
c) Health services are conveniently reachable	0.94			
4. Civic participation and employment		0.71	0.88	0.88
a) Elderly people have flexible voluntary options	0.82			
b) Flexible paid opportunities for elderly people to work	0.90			
c) Workplaces are adapted to meet the needs of elderly people with disabilities	0.81			
5. Social connectedness		0.81	0.94	0.94
a) Disconnected from the world around me	0.78			
b) No sense of togetherness	0.92			
c) Don’t feel related to anyone	0.94			
d) Losing all sense of connectedness with society	0.94			

Note: ^a^Std. factor loadings denotes standardised factor loadings; formula for ^b^average variance extracted (AVE) and ^c^composite reliability (CR) were:

$\mathrm{AVE}=\frac{\sum \text{standardised}\;{\text{factor loadings}}^{2}}{\text{Number of items}};\;$ $\mathrm{CR}=\frac{\sum {\left(\mathrm{Sum}\;\text{of standardised factor loadings}\right)}^{2}}{\left({\sum \text{standardised factor loadings}}^{2}\right)+\left(\sum 1-\text{standardised}\;{\text{factor loadings}}^{2}\right)}$

The results showed that convergent validity was achieved with all the items, with satisfactory standardised factor loadings exceeding 0.7. The AVE indicated that all latent variables had good validity with all values greater than 0.5. For the reliability assessment, all the constructs were acceptable with composite reliability (CR) and Cronbach’s alpha coefficient exceeding 0.7.

Table 5 shows the results of discriminant validity using the method of Fornell and Larcker (1981). ^47^ The constructs achieved discriminant validity, as the bold values are larger compared to the other values.

**Table 5.** Discriminant validity analysis

Age-friendly environment constructs	1	2	3	4	5
1. Communication and information	**0.89**				
2. Built environment	0.17	**0.67**			
3. Community support and health services	0.12	0.50	**0.88**		
4. Civic participation and employment	0.21	0.39	0.36	**0.71**	
5. Social connectedness	0.00	0.03	0.02	0.01	**0.81**

Note: Values in diagonal (bold) are average variance extracted (AVE) values while others are squared correlations among variables.

Second-order CFA indicated satisfactory standardised factor loading with three items exceeding 0.5 and one item near 0.5. The model was statistically fit with CMIN/df = 1.37, p-value = 0.05, GFI = 0.97, AGFI = 0.95, RMSEA = 0.03, SRMR = 0.03, NFI = 0.98, TLI = 0.99, CFI = 0.99 and ECVI = 0.39. The second-order model indicated that among the four, built environment had the highest standardised factor loading of 0.86 and the largest causal effect towards an age-friendly environment. Community support and health services followed next with a standardised factor loading of 0.80. Next was civic participation and employment with a standardised factor loading of 0.76. Communication and information, with a standardised factor loading of 0.49, had the smallest causal effect towards an age-friendly environment.

Although the cut-off point for standardised factor loading was 0.5, communication and information, with a standardised factor loading of 0.49, was not removed from the model. This was because the model achieved satisfactory model fit, indicating no further model re-specification procedure was required. Moreover, a standardised factor loading of 0.49 is very close to 0.5. It is worth noting that in the CFA presented by Paúl *et al.* (2012) ^50^ and Lee and Scott (2004), ^51^ indicators were not removed when factor loading was less than 0.5.

**Figure 2.**
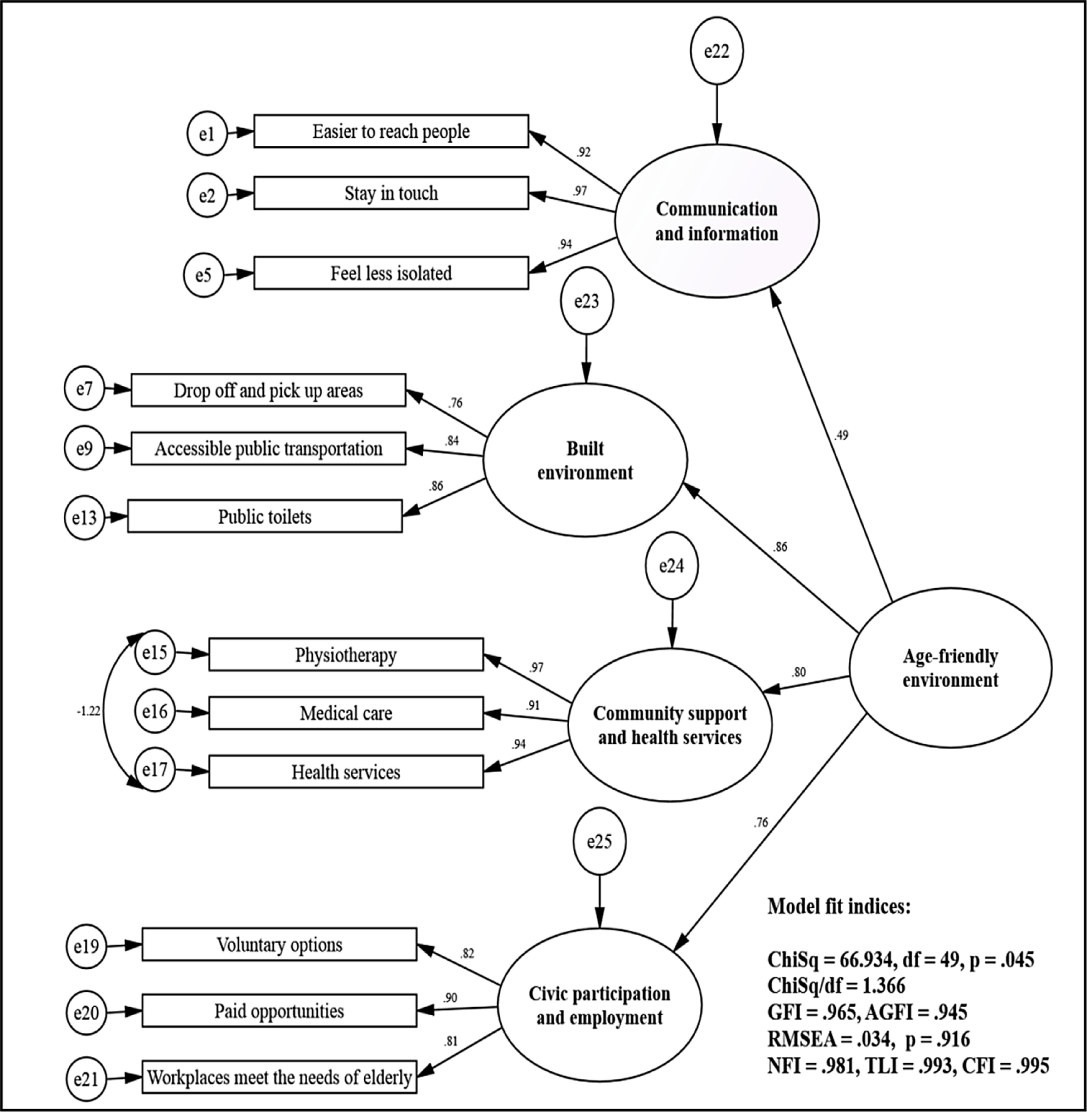
Second-order confirmatory factor analysis of age-friendly environment features. ChiSq, chi-square; df, degrees of freedom; GFI, goodness-of-fit index; AGFI, adjusted GFI; RMSEA, root mean square error of approximation; NFI, normed fit index, TLI, Tucker Lewis index; CFI, comparative fit index.

Given these results, H _1_ can be rejected. Instead of eight, only four constructs, namely built environment, community support and health services, civic participation and employment, and communication and information were confirmed as constructs of an age-friendly environment. These four constructs are in line with the characteristics of age-friendly environments described by Feldman and Oberlink (2003) ^26^ and Alley *et al.* (2007). ^27^ An age-friendly environment included supportive and accessible social services as well as safe neighbourhoods, which was supported by the findings of Alley *et al.* (2007). ^27^ The results of second-order CFA are presented in Figure 2.

Next, a measurement model was specified by allowing age-friendly environment and social connectedness features to co-vary. The initial model achieved a fairly satisfactory fit as p-value was at 0.05. Model re-specification was carried out by removal of the item “I have no sense of togetherness with my peers” from social connectedness. Removing an item from the social connectedness variable was theoretically justified as the remaining three items still had a similar underlying sense of “connectedness” (Lee & Robbins, 1995, p. 236). ^38^ Hence the final model achieved satisfactory fit with p-value = 0.08, CMIN/df = 1.22, GFI = 0.96, AGFI = 0.94, RMSEA = 0.03, SRMR = 0.05, NFI = 0.98, TLI = 0.99, CFI = 0.99.

The structural model was specified by linking age-friendly environments to social connectedness. The structural model was valid as all the items had standardised factor loadings of at least 0.5. It met satisfactory goodness of fit for all the indices with an insignificant p-value at 0.08, CMIN/df = 1.22, GFI = 0.96, AGFI = 0.94, RMSEA = 0.03, SRMR = 0.05, NFI = 0.98, TLI = 0.99 and CFI = 0.99. The model showed that age-friendly environments were found to be positively associated with social connectedness, with a standardised coefficient of 0.18.

**Figure 3.**
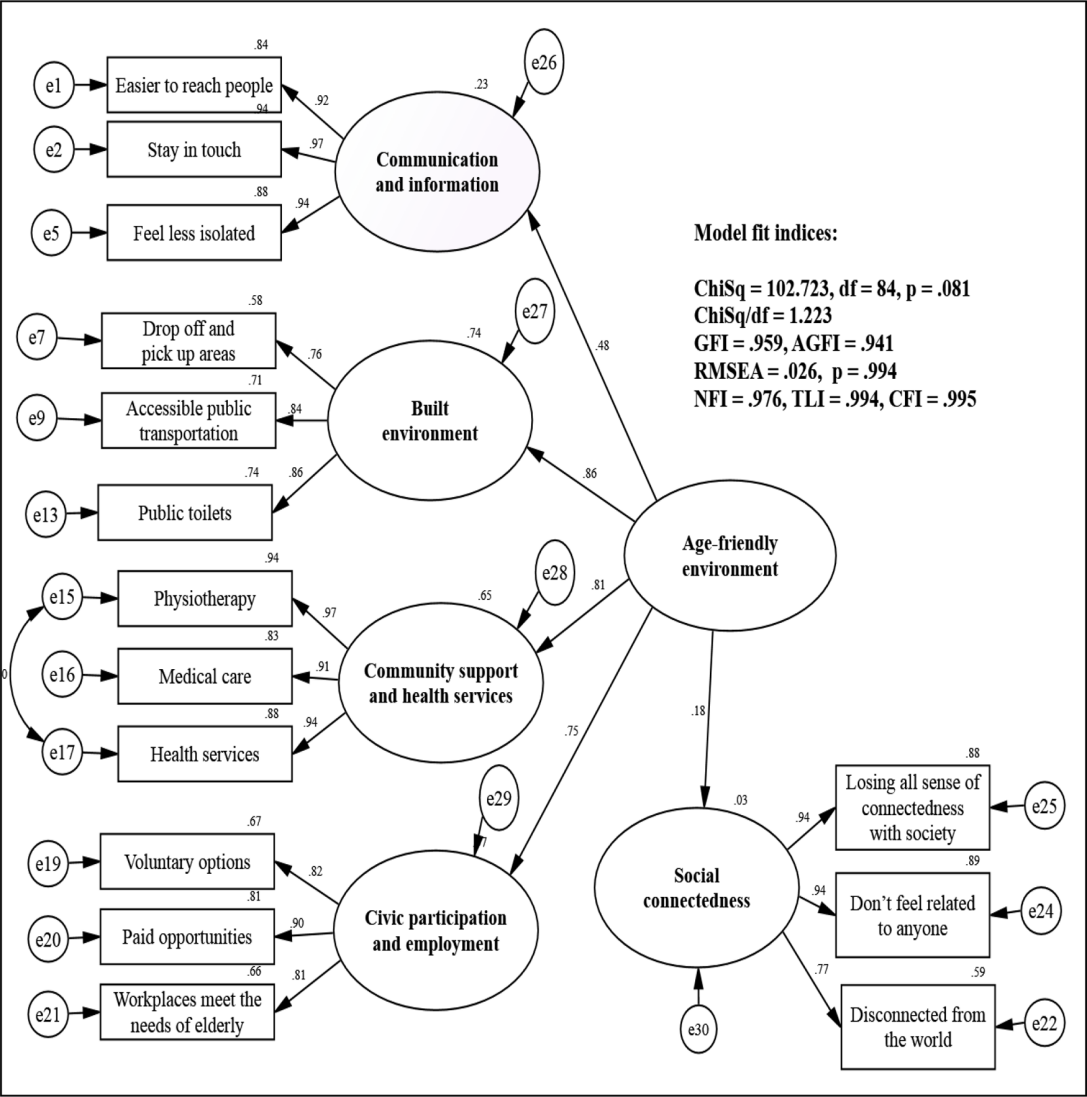
Structural model of age-friendly environment and social connectedness features. ChiSq, chi-square; df, degrees of freedom; GFI, goodness-of-fit index; AGFI, adjusted GFI; RMSEA, root mean square error of approximation; NFI, normed fit index, TLI, Tucker Lewis index; CFI, comparative fit index.

Approximately 3% of the variation in social connectedness was explained by the variation in age-friendly environment features. Hence, H _2_ was supported, indicating that age-friendly environments are positively related to social connectedness. This finding is in line with the findings of Menec *et al.* (2011) ^16^ and Emlet and Moceri (2012). ^10^ The positive relationship indicates that age-friendly environments help to promote and support social connectedness among middle-aged and older adults caring for themselves. ^16^ Middle-aged and older adults caring for themselves who were leading an active and socially connected lifestyle were able to strengthen their emotional health, improve their general well-being, and delay their ageing process. ^31^ The structural model of age-friendly environments and social connectedness is illustrated in Figure 3.

## Discussion

It was worth noting that not all the eight features of WHO’s (2007) ^12^ age-friendly cities were perceived to be significant in the Malaysia context. Built environment was found to be the most important construct of an age-friendly environment. Built environment increased protection and security for middle-aged and older adults caring for themselves when they move around and interact with the environment and community. ^16^ As an environment becomes friendly, they can participate in social activities, exercise in the park, receive adequate healthcare, go shopping, and visit family and friends. ^10^^,^^16^ A senior-friendly public transportation system was also important for their mobility. ^25^^,^^28^ Having access to community healthcare services and recreational facilities could improve their social and physical well-being, which indirectly becomes an alternative to health promotion. ^18^

Community support and health services were found to be strongly associated with age-friendly environments. These findings are consistent with Ruza *et al.* (2015) ^30^ and Lai *et al.* (2016). ^31^ Age-friendly community support and health services enable middle-aged and older adults caring for themselves to receive necessary healthcare at the lowest cost to maintain a strong sound body and mind. ^9^^,^^12^ Being healthy increases the capacity of middle-aged and older adults caring for themselves to interact with and live in their existing environment. ^21^ Therefore, healthcare and medical services found within local communities are beneficial as these adults do not solely dependent on the services provided by the state or federal government.

Civic participation and employment referred to voluntary options and paid opportunities in workplaces that take into consideration the disabilities of the ageing adults. The results indicated that middle-aged and older adults caring for themselves in Malaysia preferred to contribute and participate in society through paid or voluntary opportunities after their retirement. This result echoed the findings of Feldman and Oberlink (2003) ^26^ who indicated that financial security as well as social and civic engagement were important age-friendly features.

Fourth, the communication and information construct was found to have a weak association with age-friendly environments, which was supported by Ruza *et al.* (2015). ^30^ The result shows that communication and information obtained through the Internet assist with and ease communication among middle-aged and older adults caring for themselves, helping them to stay in touch with people, which in turn makes them feel less isolated. A possible explanation is that middle-aged and older adults caring for themselves are afraid of losing up-to-date information, news and being isolated from society. ^12^

On the other hand, social participation and respect and social inclusion were not significant constructs of an age-friendly environment. A possible reason may be that poor public transport systems and service quality in Malaysia are disabling rather than enabling and may hinder some social participation. The public transportation system in Malaysia is not well connected or friendly to older people. Poor services and inaccessible public transportation in Malaysia discouraged people from using it. ^52^

The results have implications for the Malaysian government and its related agencies to develop public policies for the welfare of its ageing population and older adults. The government must improve the living environment of the ageing population and focus on the physical environment. Furthermore, the results found that middle-aged and older adults caring for themselves were keen to participate in the labour force. Therefore, the authorities could encourage the private sectors to hire ageing workers by subsidising their training expenses or their salary. The Inland Revenue Board of Malaysia could develop schemes for income tax rebates or tax reliefs for companies that recruit older employees aged 60 years and above.

The results imply opportunities for a sustainable economy. An age-friendly workplace reduces the barriers to working opportunities for older employees. ^12^ Business organisations can renovate their workplace or design an ergonomic workplace to accommodate the physical considerations of ageing adults. The findings support the development of an age-friendly business industry within the age-friendly environment. An age-friendly business industry is a solution to economic growth and sustainability as there are opportunities for new businesses, ^16^ such as building senior-friendly housing with safety features, recreation facilities, and healthcare centres in the community. Another age-friendly business opportunity is to help middle-agers plan financially for their retirement, such as offering long-term care insurance and private retirement schemes. ^53^

## Conclusions

The overall findings indicate a simpler age-friendly environment in the Malaysian context, which evidenced only four age-friendly features instead of the eight recommended by WHO (2007). ^12^ Built environment was found to be the most important construct, implying that governments of developing countries should give priority to developing features of the built environment, such as a supportive external environment and an accessible public transportation system. In Malaysia, many of the built environment features and an age-friendly transportation system have yet to be comprehensively formed. ^29^ The poor public transportation system may affect the development of social environment features such as opportunities for civic engagement and social participation. ^28^^,^^30^

A non-probability sampling method was employed and most of the respondents were active adults caring for themselves, which may limit the generalisation of the findings. Therefore, attention should be paid when generalising the results to other developing countries or different settings. Future studies can examine the moderative effect of demographic variables such as gender and culture. An age-friendly environment index would enable the authorities to further identify areas of improvement for the benefit of the ageing population.

The results concluded that an age-friendly environment helps middle-aged and older adults caring for themselves to feel connected to the world and society as well as to feel related to anyone. Age-friendly environments help elderly people to stay active and participate in their community through accessible transportation systems and barrier-free environments. ^26^ Social connectedness can be promoted by eliminating the physical barriers that arise from the built environment. ^25^^,^^29^ Feeling connected with society, family, and friends can become a form of emotional resource. ^29^ Age-friendly environments enable elderly people to improve their social connectivity, indirectly promoting independence as well as physical and social well-being.

## Data availability

### Underlying data

Figshare: Elderly v2. https://doi.org/10.6084/m9.figshare.14870118.v4. ^55^

This project contains the following underlying data:

- Elderly pilot study.csv (raw data from pilot study)
- Elderly.csv (raw data from main study)
- Data dictionary.csv

### Extended data

Figshare: Questionnaire (age-friendly). https://doi.org/10.6084/m9.figshare.16530777.v1. ^54^

Data are available under the terms of the Creative Commons Attribution 4.0 International license (CC-BY 4.0).

## Footnotes

^1^
Individuals aged 45 to 50 are classified in the stage “entry life structure of middle adulthood” while individuals aged 60 to 65 are classified in the stage “late adult transition” ^33^ (Levinson, 1986, p. 7).
