# Supplementary material for: Modelling age-friendly environment for social connectedness: a cross-sectional study
Source: F1000Res. 2021 Sep 22;10:955. [Version 1] doi: 10.12688/f1000research.73032.1 (PMC8729032; doi:10.12688/f1000research.73032.1)
Supplement: Peer review history

# Peer review: Reviewer response for version 1

**DOI:** 10.5256/f1000research.76651.r101635

**Published:** 2021-12-22

**Authors:** Mark Ward

**Affiliations:** 1 Trinity College Dublin, Dublin, Ireland

This is a very detailed and comprehensive piece of work which adds to the literature on age-friendly environments. I do however have some suggestions and queries that the authors might consider addressing. The most important of these relate to sampling and the choice of sub-samples for the factor analysis and SEM analyses.

 When describing Bronfenbrenner's ecological theory, it is important to also include the fifth system, the chronosystem, particularly when describing the social environment of older adults. After all, age(time) is a crucial factor to consider. Time is also important when considering things like the built environment as urban areas developed at different times may have more or less age friendly features. This is alluded to in paragraph 5 page 4 "In the application of ecological frameworks...".

 As previous reviews have mentioned, there is a extensive more recent literature that could be referred to. For example, see DOI: 10.1080/13607863.2019.1652246 and work referred to within there.

 The introduction and literature review are quite repetitive and this is not helped by the listing of previous literature. Rather than arranging these sections by study, why not arrange it by topic and reference whatever number of studies within each section?

 Last paragraph page 5. I am not familiar with the term 'reflective model'. What does it mean? The same paragraph. This is a needlessly verbose way of saying something very simple. That is, you have 8 domains derived from 37 (I think) indicators.

 The following sentence does not belong in the study design section "Middle-aged and older adults have witnessed death in their family and of their peers; thus, they may be compelled to improve their quality of life while they still have time". 

 I don't understand why the factor analysis included only 82 of the 402 participants? Similarly, why only include 320 in the SEM model? Could the full sample not have been used for both? In any event, please explain how/why these numbers were arrived at, beyond the fact that they are the minimum sample size according to your power calculation.

 I am not convinced that the sampling procedure was actually purposive. The purposive part appears to be the 272 recruited at senior activity centres. Sampling from local community areas strikes me more as convenience sampling. If truly purposive sampling, then what were the inclusion & exclusion criteria? What is simply age?

 Much is made in some places of the distinction between middle-aged and older-aged but I can not see where this is considered analytically. While the study might not be powered to do so, did the authors consider stratifying the SEM model by these two age groups?

 Is is reasonable to suspect that evaluations of "the level of importance of age-friendly environment features" are partly informed by where people actually live. Therefore, I wonder what if results varied at all according to the seven locations from within which participants were recruited. Again, there might not be adequate power to assess this fully but it should at least be possible to adjust the standard errors for clustering in the SEM model.

 When referring to the SEM 'research model', do you mean 'measurement model'?

 Page 8: "Bartlett’s test of sphericity was significant with p = 0.00". A p value can never be truly zero. Please report this as <0.001.

 Finally, "Approximately 3% of the variation in social connectedness was explained by the variation in age-friendly environment features. Hence, H2 was supported, indicating that age-friendly environments are positively related to social connectedness". This is a very small effect size so the effect the age-friendly domains included here have on social connectedness are not over-stated. Do the authors have any thoughts on what other important factors there might be.

Is the work clearly and accurately presented and does it cite the current literature?

Partly

If applicable, is the statistical analysis and its interpretation appropriate?

Partly

Are all the source data underlying the results available to ensure full reproducibility?

Yes

Is the study design appropriate and is the work technically sound?

Yes

Are the conclusions drawn adequately supported by the results?

Yes

Are sufficient details of methods and analysis provided to allow replication by others?

Partly

Reviewer Expertise:

Ageing

I confirm that I have read this submission and believe that I have an appropriate level of expertise to confirm that it is of an acceptable scientific standard, however I have significant reservations, as outlined above.

## References

[1] : Age-friendly environments and psychosocial wellbeing: a study of older urban residents in Ireland. *Aging Ment Health* .24(12) : 10.1080/13607863.2019.1652246 2022-2033 10.1080/13607863.2019.1652246 31402677

# Peer review: Reviewer response for version 1

**DOI:** 10.5256/f1000research.76651.r97564

**Published:** 2021-11-04

**Authors:** Peik Foong Yeap

**Affiliations:** 1 University of Newcastle, Singapore, Singapore

This paper adapted the WHO 2007 framework of features of age-friendly cities to examine age-friendly environment constructs and their linkages with social connectedness from the perspective of Malaysian middle-aged and older adults caring for themselves. Structural equation modelling was performed with 320 samples. The structural model indicated four features i.e. built environment, community support and health services, civic participation and employment as well as communication and information enable the ageing population to improve their connectedness with society in Malaysia. The result of the study was in line with the present literature in which an age-friendly environment supports and cultivates positive social relationships and connectivity. Various implications for the Malaysian government and its related agencies to develop public policies for the welfare of its ageing population and older adults are discussed.

 This study is interesting and timely to discuss the age-friendly environment for social connectedness among ageing population in Malaysia. However, is there any reason/s why Malaysia has not adopted the WHO (2007) global age-friendly cities guide as mentioned in the Introduction (para. 7, line 1)? 

 Bronfenbrenner’s ecological theory (1977) discusses the structures of microsystem, mesosystem, exosystem and macrosystem. There is a need to further explain exosystem and macrosystem, and how these four systems are interrelated in the literature review section.

 Where was Alley *et al.'s* (2007) study of the elder-friendly communities using qualitative methods being carried out? Similar to Ruza *et al.* (2015) and Rogers and Mitzner (2017) studies respectively. More clarifications are needed.

 The study collected data from 402 respondents from middle-aged and older adults caring for themselves in the seven Malaysian states. Among these data, 272 responses were collected at senior activity centers in the selected seven states of Malaysia. Meanwhile, 130 responses were data gathered from local community areas. The authors need to explain further the control mechanism that have been used to avoid duplication of data collection in the "Data collection" section.

 It would be good to add more current literature and findings to enhance the robustness of the study.

Is the work clearly and accurately presented and does it cite the current literature?

Partly

If applicable, is the statistical analysis and its interpretation appropriate?

Yes

Are all the source data underlying the results available to ensure full reproducibility?

Yes

Is the study design appropriate and is the work technically sound?

Yes

Are the conclusions drawn adequately supported by the results?

Yes

Are sufficient details of methods and analysis provided to allow replication by others?

Yes

Reviewer Expertise:

Organizational inclusiveness and behavior, strategic management, teaching and learning

I confirm that I have read this submission and believe that I have an appropriate level of expertise to confirm that it is of an acceptable scientific standard.

# Peer review: Reviewer response for version 1

**DOI:** 10.5256/f1000research.76651.r95040

**Published:** 2021-10-04

**Authors:** Amy Yeo Chu May

**Affiliations:** 1 Department of Management, Tunku Abdul Rahman University College Faculty of Accountancy Finance and Business, Kuala Lumpur, Malaysia

This study investigates primarily on age-friendly environment and social connectedness in the context of a developing country. The authors have been able to demonstrate a well-designed research methodology which encumbrances the essential areas such as gap identification, research objectives, literature review right up to the end results. Well done for this!

**The authors may want to consider tweaking the following areas for further improvement:**

**Literature review**:

 Some literature concerning the old-age related studies have been examined in the authors’ country, as cited (Lai *et al*., 2016). The authors also have carefully examined some studies taken place in other part of the globe which is laudable! However, current literature (2019-2021) on old-age related studies at ‘national level’ seem to be less used in this study. I suggest the authors lay down the relevant literatures (national/international) before leading to the hypothesis I. The breakdown of each construct/variable (eg: outdoor sparse and building, transportation etc.) and the aged-friendly environment should be supported by relevant prior studies with succinct justifications.

**Research Methods**:

 This section was well articulated in terms of the appropriate research design, data collection, sampling, design of questionnaire and statistical tools that are suited to the current study.

**Data analysis and results:**

 Several statistical tools such as reliability and validity test as well as AVE were performed on the raw sample of 402. However, why was EFA only performed on 82 responses? It would be advisable for the authors to rationalise it for the benefit of potential readers who may not have the statistical knowledge. In other words, more interpretations are needed to make the statistical results more comprehensible.

**Conclusion and implications**:

 The authors concluded by highlighting only four age-friendly features are applicable in the context of Malaysia instead of eight recommended by WHO (2007). With this, the government of the country would have to consider putting up more built environment features and age-friendly transportation system. What about the other two features? Are they not as important as built environment and transportation? Basing on the outcome of this study, apart from the government intervention and support in ensuring an age-friendly environment and social connectedness, how about private sector involvement/participation so as to complement and supplement the government’s effort? Hence, to add value to the findings, I suggest the authors include the practical and theoretical implications arising from the results of the study.

Is the work clearly and accurately presented and does it cite the current literature?

Partly

If applicable, is the statistical analysis and its interpretation appropriate?

Yes

Are all the source data underlying the results available to ensure full reproducibility?

Yes

Is the study design appropriate and is the work technically sound?

Yes

Are the conclusions drawn adequately supported by the results?

Partly

Are sufficient details of methods and analysis provided to allow replication by others?

Partly

Reviewer Expertise:

Generally on social media related areas but specifically more on social and emotional competences as well as general corporate governance

I confirm that I have read this submission and believe that I have an appropriate level of expertise to confirm that it is of an acceptable scientific standard, however I have significant reservations, as outlined above.
